# Curcumin Nanoformulation for Cervical Cancer Treatment

**DOI:** 10.1038/srep20051

**Published:** 2016-02-03

**Authors:** Mohd S. Zaman, Neeraj Chauhan, Murali M. Yallapu, Rishi K. Gara, Diane M. Maher, Sonam Kumari, Mohammed Sikander, Sheema Khan, Nadeem Zafar, Meena Jaggi, Subhash C. Chauhan

**Affiliations:** 1 Department of Pharmaceutical Sciences and Center for Cancer Research, University of Tennessee Health Science Center, Memphis, Tennessee, 38163, USA; 2 Cancer Biology Research Center, Sanford Research, Sioux Falls, South Dakota, 57104, USA; 3 Department of Pathology, University of Tennessee at Memphis, Memphis, TN, USA

## Abstract

Cervical cancer is one of the most common cancers among women worldwide. Current standards of care for cervical cancer includes surgery, radiation, and chemotherapy. Conventional chemotherapy fails to elicit therapeutic responses and causes severe systemic toxicity. Thus, developing a natural product based, safe treatment modality would be a highly viable option. Curcumin (CUR) is a well-known natural compound, which exhibits excellent anti-cancer potential by regulating many proliferative, oncogenic, and chemo-resistance associated genes/proteins. However, due to rapid degradation and poor bioavailability, its translational and clinical use has been limited. To improve these clinically relevant parameters, we report a poly(lactic-*co*-glycolic acid) based curcumin nanoparticle formulation (Nano-CUR). This study demonstrates that in comparison to free CUR, Nano-CUR effectively inhibits cell growth, induces apoptosis, and arrests the cell cycle in cervical cancer cell lines. Nano-CUR treatment modulated entities such as miRNAs, transcription factors, and proteins associated with carcinogenesis. Moreover, Nano-CUR effectively reduced the tumor burden in a pre-clinical orthotopic mouse model of cervical cancer by decreasing oncogenic miRNA-21, suppressing nuclear β-catenin, and abrogating expression of E6/E7 HPV oncoproteins including smoking compound benzo[a]pyrene (BaP) induced E6/E7 and IL-6 expression. These superior pre-clinical data suggest that Nano-CUR may be an effective therapeutic modality for cervical cancer.

Cervical cancer is one of the most common and deadly cancers among women worldwide and is associated with persistent Human Papillomavirus (HPV) infection^1^. Only a small subset of women with chronic HPV infection progresses to develop the disease^2^. Additional factors are needed to acquire an immortal phenotype and to further advance towards malignant and invasive phenotypes^3^,^4^. In addition to HPV infection, cigarette smoking and smoke carcinogen (benzo[a]pyrene, BaP), are known risk factors associated with cervical cancer^2^,^5^. Viral morphogenesis is increased subsequent to BaP treatment of cells infected with the high-risk HPVs, 31, 16 and 18 in organotypic raft cultures derived from a cervical intraepithelial neoplasia type I cell line^6^. Moreover, micro RNAs (miRNAs), small noncoding RNAs that regulate the expression of protein-coding genes, also play an important role in the development of carcinogenesis. Resistance to chemo/radio-therapies with prolonged treatment, resulting in an invasive form of cancer, requires the development of novel therapeutic modalities to conquer chemo-resistance and improve the overall life expectancy of patients.

Curcumin (CUR) is a natural polyphenol compound that is derived from the rhizome of the medicinal plant *Curcuma longa Linn*. It has been widely used in traditional Indian medicine for its efficacy against inflammation, respiratory diseases and other disorders^7^,^8^. Due to its anti-inflammatory and anti-carcinogenic qualities, it has also been extensively studied in the field of cancer therapeutics. CUR has shown dose-dependent chemopreventive and chemotherapeutic effects in a number of studies and pre-clinical trials^9^,^10^. Curcumin exhibits cytotoxic effects in cervical cancer cells in a concentration-dependent and time-dependent manner and its activity was found to be higher in HPV infected cells^11^. Curcumin has been proven to downregulate HPV18 transcription by selectively inhibiting AP-1 activity, which reverses the expression dynamics of c-fos and fra-1 in cervical cancer cells^11^. Superior inhibitory action of curcumin against cervical cancer cells^12^,^13^,^14^ was due to the inhibition of telomerase activity, Ras, and ERK signaling pathways, cyclin D1, COX-2 and iNOS activity, and the mitochondrial pathway. Interestingly, curcumin acted upon multiple targets and due to pretreatment was in turn able to revert the proliferative effects of cervical cancer cells. A recent proteomic study suggests that curcumin induces significant changes in tumor-related proteins that are associated with cell metabolism, cell cycle, and carcinogenicity in HeLa cells^15^. Additionally, curcumin acts as a sensitizer for chemotherapy and radiation in cervical cancer therapy. We reported a detailed cellular suppressive mechanistic role of curcumin in a three-dimensional cervical cancer raft culture system^5^. Our study demonstrated that curcumin inhibited cell motility, induced apoptosis, decreased the expression of HPV oncoproteins, and restored tumor suppressor proteins. At present, about 55 clinical trials are listed on clinicaltrial.gov related to curcumin and cancer therapy (as of December 2, 2015), which suggest its translational and clinical potential. Furthermore, it has demonstrated no toxicity to healthy organs at higher doses such as 8 g/day in clinical trials^16^. However, it suffers from very low systemic bioavailability, poor pharmacokinetics, poor absorption ability, high metabolic rate, inactivity of metabolic products, together with rapid elimination and clearance from the body^17^,^18^,^19^. Some studies have shown that a trace amount of CUR was detected in the serum of humans when 4–12 g/day of CUR was administered^17^,^20^. Although curcumin has inspired considerable interest for its extensive physiological activities, its poor bioavailability restricts its clinical translation.

Nanoparticle technology provides an effective way to deliver anti-cancer drug(s) into tumors^21^. To circumvent curcumin’s inherent issues, our lab has developed a curcumin nanoparticle formulation (Nano-CUR), (Fig. 1A,B) based on poly(lactic-*co*-glycolic acid) (PLGA), an FDA approved polymer. This formulation has shown to be effective for improved therapeutic effects in metastatic ovarian and breast cancer cells^22^. The objective of this study was to implement this efficient Nano-CUR formulation to achieve improved anti-cancerous effects on cervical cancer. Our results indicated that Nano-CUR effectively inhibited growth of cervical cancer cells (Caski and SiHa), induced apoptosis, and arrested cell cycle in the G1-S transition phase. Moreover, Nano-CUR formulation effectively reduced the tumor burden in NOD SCID gamma (NSG) orthotopic xenograft mouse model. Additionally, Nano-CUR treatment caused a marked decrease in the levels of miRNA-21 (an onco-miRNA associated with chemo-resistance), in *in vitro* and *in vivo* models^23^,^24^, and enhanced the expression of miRNA-214 (a tumor suppressor)^25^,^26^, when compared to free CUR, besides decreasing the levels of IL-6 (Interleukin-6) cytokine expression which was found to be enhanced with BaP treatment. Altogether, these results suggest that Nano-CUR provides a beneficial approach for a rational strategy to widen the chemo-preventive and therapeutic modality for the overall management of cervical cancer.

## Results

### Internalization of Nano-CUR in cervical cancer cells

**Figure 1:**
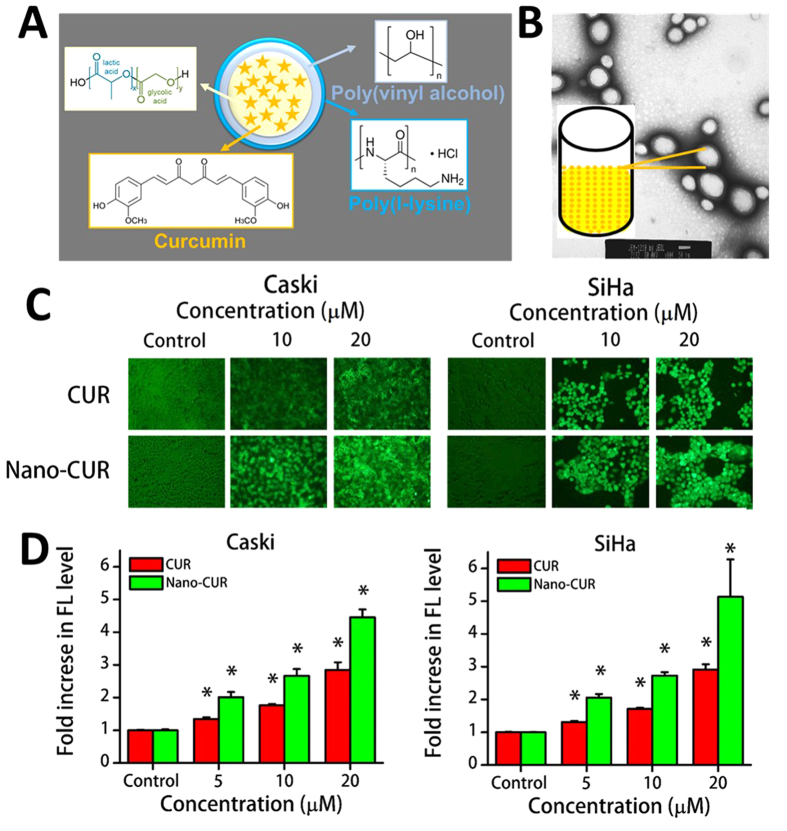
Nano-CUR improves cellular accumulation of curcumin in cervical cancer cells. (**A**) Structural components of Nano-CUR formulation. (**B**) Representative transmission electron microscopy image of Nano-CUR particles. (**C**) Fluorescent images of live cells showing increased uptake of CUR/Nano-CUR with increase in dose. Original magnification 200X. (**D**) Samples were analyzed by flow cytometry for cellular uptake. Fold change in mean fluorescence normalized to the respective vehicle controls. Error bars show SEM; N = 3, average of 3 independent experiments, done in triplicate; *p < 0.05.

The efficient internalization of drug molecules contributes to their improved anti-cancer activity. To examine this phenomenon, we selected two HPV infected cervical cancer cell lines (Caski and SiHa) as our *in vitro* model. SiHa cells have 1–2 copies of integrated HPV 16^27^. Caski cells are a robust model of HPV because they contain about 600 copies of integrated HPV 16^28^. Fluorescence microscopy and flow cytometry techniques were used to examine the internalization patterns of CUR and Nano-CUR. The internalization was found to increase with an increase in the concentration of CUR and Nano-CUR in Caski and SiHa cervical cancer cells (Fig. 1C,D). Fluorescence microscopy analysis revealed that cells incubated with free CUR exhibited presence of CUR in the periphery and cytoplasm (Fig. 1C). Whereas, in the case of Nano-CUR, internalization was more efficient by endocytosis process due to smaller size, and therefore, greater accumulation of CUR was observed on the cell membrane and cytoplasm (Fig. 1C). This could be due to the result of greater interaction of Nano-CUR with the cell surface. This effect might aid internalization of the drug. A similar improved uptake of Nano-CUR was observed in the flow cytometry analyses (Fig. 1D).

### Nano-CUR inhibits growth of cervical cancer cells

**Figure 2:**
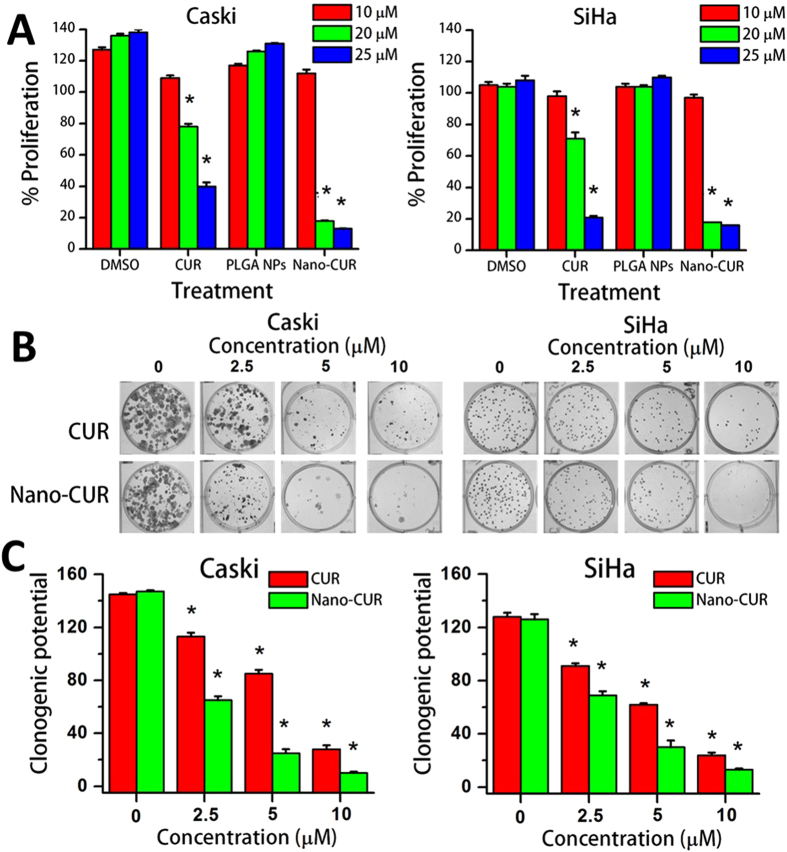
CUR/Nano-CUR inhibit proliferation and clonogenic potential of cervical cancer cells. (**A**) Proliferation was determined using MTS method with Caski and SiHa cell lines. Results were normalized to control wells treated with appropriate amounts of vehicle, DMSO for CUR and PLGA for Nano-CUR. Error bars show SEM; N = 3, average of 3 independent experiments, done in replicates of 6; *p < 0.05. (**B**,**C**) Clonogenic potential was performed with Caski and SiHa cells. (**B**) Representative colony formation images of cervical cancer cells upon treatment. (**C**) Inhibitory effect of treatments measured by colony counting. Graphs represent average of three independent experiments, each replicated three times; *p < 0.05.

To assess the anti-cancer potential of CUR and Nano-CUR in cervical cancer, Caski and SiHa cells were treated with 10, 20, and 25 μM CUR or Nano-CUR for 48 hrs. Both cell lines showed an inhibitory effect of CUR/Nano-CUR on cellular proliferation, especially at higher concentrations (20 and 25 μM) (Fig. 2A). To determine the long-term effect of CUR/Nano-CUR on the growth of cervical cancer cells, we performed a colony formation assay (Fig. 2B,C). Nano-CUR was found to be more effective as compared to free CUR in reducing the clonogenic ability of cervical cancer cells (Fig. 2B,C).

### Nano-CUR inhibits cell cycle and induces apoptosis

**Figure 3:**
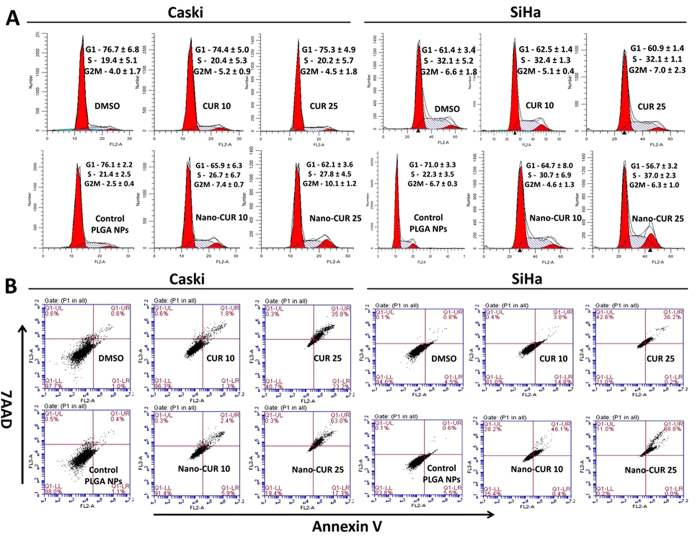
Effect of CUR and Nano-CUR on cell cycle and apoptosis in cervical cancer cells. (**A**) CUR/Nano-CUR treatment arrests Caski and SiHa cells at the G1-S transition. Cervical cancer cells were treated with CUR/Nano-CUR for 24 hrs. Samples were analyzed by flow cytometry for cell cycle analysis. Error show SEM; N = 3, average of 3 independent experiments, done in triplicate. (**B**) CUR/Nano-CUR treatment induces apoptosis in Caski cervical cancer cell line. Flow cytometry was done to detect the early apoptosis (Annexin V +ve) or late apoptosis (Annexin V +ve and 7AAD +ve).

To elaborate functional effects of CUR and Nano-CUR on cervical cancer, Caski and SiHa cells were treated with varied concentrations of CUR, Nano-CUR and their respective vehicle controls (DMSO and PLGA NPs) for 24 hrs as described in Methods. After 24 hrs, cells were trypsinized and processed for flow cytometry analyses for cell cycle and apoptosis. CUR and Nano-CUR were found to arrest Caski and SiHa cells in the G1-S transition phase (Fig. 3A). The effect was more pronounced at higher concentrations. Nano-CUR was found to be more effective in comparison to free CUR in inducing apoptosis in Caski and SiHa cells (Fig. 3B).

### Nano-CUR effectively inhibits tumor growth in NOD SCID gamma (NSG) orthotopic xenograft mouse model

**Figure 4:**
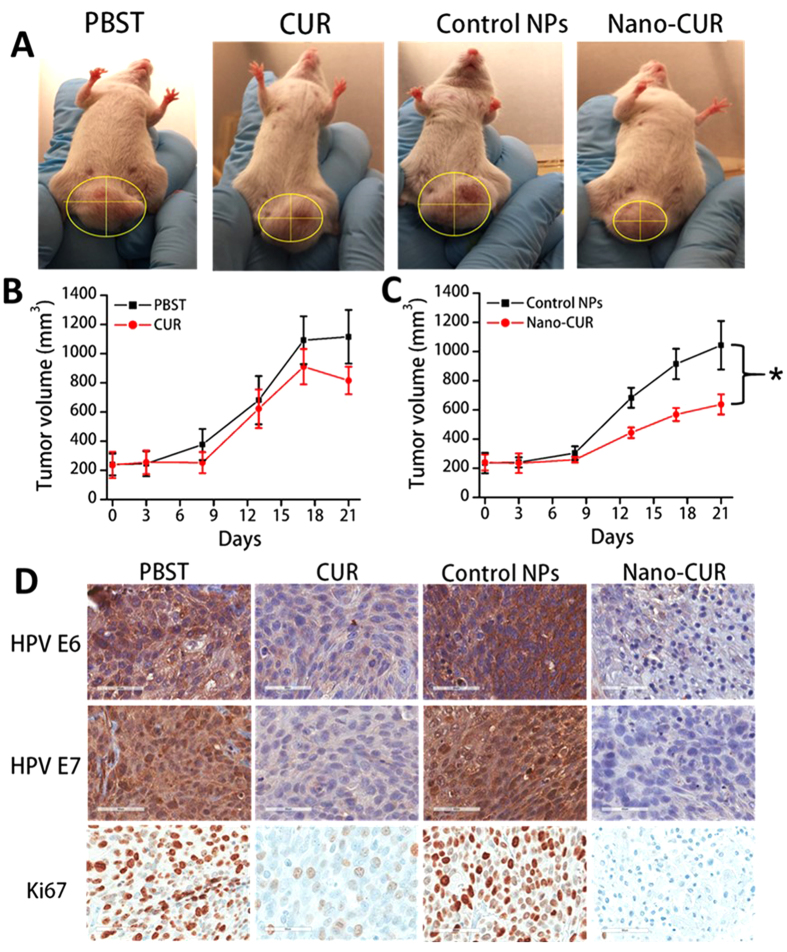
Nano-CUR significantly reduces tumor growth and oncogenic HPV E6/E7 and Ki67 proteins in cervical cancer orthotopic mouse model. Tumor formation in female nude mice by injecting 4 million Caski cells. (**A**–**C**) Nano-CUR was able to reduce the tumor burden by a considerable amount as compared to free CUR. Error bars show SEM; N = 6; *p < 0.05. (**D**) Immunohistochemical (IHC) analysis showed that CUR and Nano-CUR were effective in suppressing the expression of HPV oncogenic proteins E6 and E7 and cell proliferation marker Ki67 in mice. Original magnification 400X.

An orthotopic mouse model was developed and used to assess the efficacy of CUR and Nano-CUR to inhibit tumor growth in NSG mice. For that, Caski cells were injected directly into the cervix of NSG mice. Following tumor development (~200 mm^3^), the mice were administered with intra-tumoral injections of CUR, Nano-CUR and their respective vehicle controls (PBST and PLGA NPs, respectively). Figure 4A illustrates the mice treated with various treatment groups. The tumor volumes were measured and presented on the days shown in Fig. 4B,C. At the termination of the experiment, mice were euthanized, interestingly CUR and Nano-CUR treatment (Fig. 4B,C) demonstrated inhibition of tumor growth over their respective control groups. However, Nano-CUR treatment was found to be more effective than all other treatment groups. Average tumor volume in all groups are as follows: Nano-CUR (637 ± 68 mm^3^) <CUR (816 ± 94 mm^3^) <PLGA NPs (control NPs; 1042 ± 166 mm^3^) <PBST (1115 ± 184 mm^3^) (Fig. 4B,C).

### Nano-CUR effectively represses the expression of HPV oncoproteins in tumor tissues

HPV oncoproteins, E6/E7, initiate the dysregulation of cellular proliferation and apoptotic mechanisms by targeting p53 and retinoblastoma (Rb) tumor suppressor proteins, respectively^29^. This leads to oncogenesis and the development of cervical carcinoma. Also, higher expression of Ki67, a commonly used marker for cell proliferation, is associated with high-grade cervical intraepithelial neoplasia (CIN)^30^. Immunohistochemistry done on the tissue slides made from the orthotopically generated mice tumors treated with CUR/Nano-CUR, and their respective controls, revealed that Nano-CUR was more effective in suppressing E6/E7 and Ki67 expression levels in mice tumors when compared with free CUR (Fig. 4D).

### Nano-CUR efficiently modulated BaP induced expression of miRNAs in cervical cancer cells

**Figure 5:**
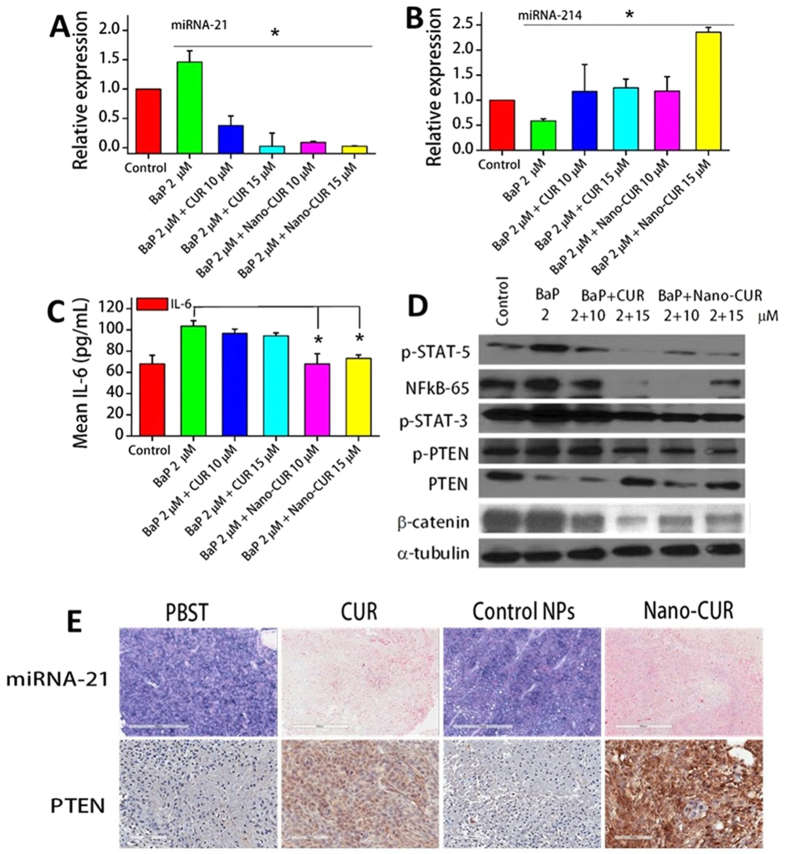
Effect of BaP, CUR and Nano-CUR on miRNAs, cytokine IL-6 expression and related pathways in Caski cells. BaP was found to: (**A**) increase the expression of oncogenic miRNA-21, and (**B**) decrease the expression of tumor suppressor miRNA-214. Nano-CUR was found to be more effective than free CUR in mitigating the oncogenic effects of BaP, by decreasing miRNA-21 and increasing miRNA-214. (**C**) BaP increased the expression of oncogenic IL-6, which was decreased effectively by CUR and Nano-CUR. (**D**) CUR and Nano-CUR restore anti-survival pathways affected by BaP by suppressing oncogenic transcription factors and restoring tumor suppressor PTEN. Control-DMSO + PLGA; BaP-Benzo(a)pyrene; CUR; Nano-CUR. miRNA expression was assessed through qRT-PCR using Taqman primers and IL-6 expression was analyzed using a cytokine assay kit. Western blotting was done using commercially available antibodies. The experiments were done in triplicate. Error bars show SEM; N = 3; all concentrations are in μM; *p < 0.05. (**E**) ISH *(In situ* hybridization) of miRNA-21 and IHC of PTEN in mice tumor tissues treated with CUR and Nano-CUR. Blue marks correspond to miRNA-21 and brown marks correspond to PTEN expression. ISH and IHC were conducted according to the manufacturer’s protocol for FFPE tissues of control and treated orthotopic mice to detect the expression of miRNA-21 and PTEN. Original magnification 200X.

As mentioned earlier, in addition to HPV infection, cigarette smoking and smoke carcinogens, such as benzo[a]pyrene (BaP), are known risk factors for cervical cancer. Our prior work has shown that BaP increased the level of HPV oncoproteins, E6/E7^5^. Thus, Caski cells were treated with BaP alone and in combination with CUR and Nano-CUR. BaP was found to increase the expression of oncogenic miRNA-21 and decrease the expression of the tumor suppressor miRNA-214 which was abrogated by CUR/Nano-CUR treatment. This indicates that CUR and Nano-CUR treatments can mitigate the oncogenic effects of BaP by decreasing the expression of miRNA-21 and increasing the expression of the tumor suppressor miRNA-214. Nano-CUR treatment however, was found to be more effective than free CUR in inducing these effects (Fig. 5A,B).

### Nano-CUR inhibits BaP induced IL-6 expression in cervical cancer cells

Previous studies have shown that the IL-6 induces the expression of miRNA-21 in cancer cells^31^. Thus, we sought to investigate the effect of BaP on IL-6 in Caski cells. BaP was found to increase the IL-6 expression as compared to its vehicle control. CUR and Nano-CUR were observed to decrease the oncogenic effects of BaP by decreasing the expression of IL-6. Again, Nano-CUR was more effective than free CUR at similar concentrations in decreasing IL-6 expression (Fig. 5C).

### Restoration of anti-survival pathways affected by BaP through Nano-CUR

miRNA-21 is associated with several cancers and involved in chemoresistance and metastasis. It is also responsible for activating a number of pro-survival pathways^31^,^32^. To determine mechanisms that are involved in enhanced expression of miRNA-21, we investigated the modulations of several transcription factors and proteins that are related to miRNA-21. Western blot studies indicated that BaP treatment induced activation of transcription factors STAT-3 and STAT-5 (i.e., increased the phosphorylated form of STAT-3 and STAT-5), and NF-kB (Fig. 5D). BaP was also responsible for decreasing the expression of the anti-survival phosphatase PTEN, a direct target of miRNA-21^33^,^34^, in addition to increasing the inactive form of PTEN, phospho-PTEN (p-PTEN)^35^,^36^. CUR and Nano-CUR were observed to mitigate the oncogenic effects of BaP by reducing the expression of phosphorylated forms of STAT-3 and STAT-5, and decreasing NF-κB. Moreover, they also increased the expression of PTEN and reduced the inactivated form of PTEN, p-PTEN (Fig. 5D). CUR and Nano-CUR were also instrumental in decreasing the cytoplasmic amount of β-catenin, which is a direct target of miRNA-214^32^,^37^.

### *In vivo* assessment of miRNA-21 and its direct target, PTEN, in mice tissues treated with CUR/Nano-CUR

To examine the *in vivo* effects of CUR and Nano-CUR on miRNA expression, we investigated the expression of miRNA-21 in mice tumors treated with CUR, Nano-CUR and their respective vehicle controls. *In situ* hybridization of miRNA-21 in mice tumor tissues showed that CUR and Nano-CUR effectively reduced miRNA-21 expression as compared to their respective controls. Additionally, immunohistochemical analysis showed that CUR and Nano-CUR significantly enhanced the expression levels of PTEN, which is a direct target for miRNA-21 and a well-known tumor suppressor (Fig. 5E).

### Nano-CUR reduces BaP and IL-6 enhanced migration of cervical cancer cells

**Figure 6:**
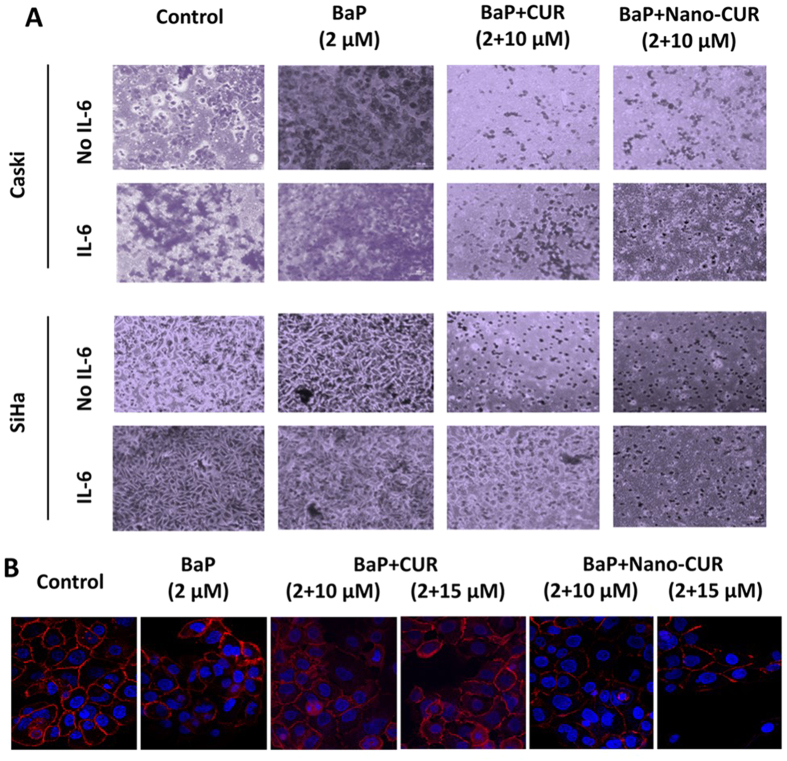
Effect of CUR and Nano-CUR on the suppression of BaP and IL-6 induced cellular migration. (**A**) CUR and Nano-CUR were able to suppress BaP and IL-6 induced cellular migration in cervical cancer cells. (**B**) Inhibition of enhanced nuclear translocation of β-catenin by BaP through CUR and Nano-CUR. For this study, 80,000 Caski cells were seeded overnight in 4-well chamber slides, then treated with BaP alone and in combination with CUR/Nano-CUR for 24 hrs. Subsequently, they were fixed and processed for immunostaining using anti β-catenin Ab. Confocal images are shown for nuclear staining (Blue for DAPI) and β-catenin (red for Cy3), using a Nikon confocal microscope. Original magnification 200X.

Previous studies have shown that BaP enhances cell motility through different mechanisms in cancer cells^38^,^39^,^40^. BaP alone was observed to increase cell migration in Caski and SiHa cells. IL-6 further enhanced the motility of the cells in combination with BaP. CUR and Nano-CUR were found to effectively reduce BaP and IL-6 mediated enhanced cellular motility of these cells (Fig. 6A).

### Nano-CUR suppresses BaP induced nuclear translocation of β-catenin

β-catenin is a key mediator of Wnt signaling, and its deregulation, resulting in its nuclear accumulation, which in turn can cause cancer progression and metastasis^41^. In normal cells, β-catenin is anchored at the plasma membrane with E-cadherin, and is maintained at low levels in the cytoplasm by an APC/axin-dependent degradation complex. Mutations occurring in β-catenin itself or its degradation complex proteins result in the inability to degrade β-catenin, which leads to the increase of protein levels of β -catenin that accumulate in the nucleus^42^,^43^,^44^. Thus it binds to the members of LEF-1/TCF family of transcription factors, subsequently resulting in stimulation of gene expression and production of proteins involved in cell transformation^45^,^46^. Therefore, persistent nuclear localization of β-catenin is the key initiating and driving event for transformation of normal (non-tumor) cell to tumor cell. Treatment with BaP resulted in an increase in the nuclear translocation of β-catenin in comparison to control as evidenced through confocal microscopy (Fig. 6B). Nano-CUR treatment was found to be more effective than free CUR, especially at higher concentrations, in reducing nuclear β-catenin accumulation and enhancing β-catenin translocation to the plasma membrane.

## Discussion

Cervical cancer is the third most common cancer in women worldwide^47^. Invasive cervical cancer and the precursor lesions are the result of persistent infection by oncogenic HPVs, predominantly HPV types 16, 18, 31, etc.^48^. However, HPV infection only is not enough to immortalize and transform the epithelial cells of the host. Additional co-factors are needed to acquire an immortal phenotype and to further develop into a blatant malignant and invasive phenotype^3^,^4^. In addition to HPV infection, cigarette smoking is a known risk factor with smoke carcinogens such as benzo[a]pyrene (BaP)^2^,^5^. BaP levels have been found to be elevated in the cervical mucus of women who smoke^49^. BaP has been shown to stimulate high levels of the viral oncoproteins E6 and E7, besides enhancing virion synthesis in cell lines prolifically infected with HPV^5^. Furthermore, mixtures containing BaP, such as cigarette smoke condensate, have been shown to induce remarkable microRNA alterations in rodent lungs and in *in vitro* human bronchial models^50^,^51^.

Conventional cytotoxic chemotherapy/radiation therapy in patients with recurrent and metastatic cervical cancer is restricted due to the eventual development of drug resistance and systemic toxicity. Therefore, it is imperative to implement biologically safe and effective natural compounds as anti-cancer and chemopreventive drugs that have already been widely used in humans and have fewer systemic side effects. One such promising molecule is curcumin, a natural polyphenol compound found in turmeric. This molecule is widely studied in the literature ( > 8,350 peer-reviewed articles, http://www.ncbi.nlm.nih.gov/pubmed/?term=curcumin, search resulted on December 02, 2015) and has been used in more than 125 clinical trials (https://clinicaltrials.gov/ct2/results?term=curcumin&Search=Search, search resulted on December 02, 2015). This molecule has a number of pharmacological activities, including anti-oxidant and anti-mutagen properties, and can inhibit the development of carcinogenesis. In our previous report, we demonstrated the anti-cancer effects and reversion of BaP-induced oncoproteins with curcumin treatment in cervical cancer cells. Based on curcumin’s excellent pleiotropic properties, its clinical implication that includes cancer treatments has increased significantly in recent years. However, its inherent bioavailability and rapid degradation characteristics restrict its use in clinical settings. Therefore, our current study utilized a novel strategy to treat cervical cancer with a curcumin nanoformulation, i.e., Nano-CUR (Fig. 1A,B).

Our data, for the first time, demonstrated significant accumulation of curcumin in cervical cancer cells when we use the Nano-CUR formulation (Fig. 1C,D). This indicates that curcumin nanoformulation uptake is favored by superior endocytosis due to its smaller size. A similar enhanced cellular uptake was observed even in other types of cancer cells^22^. Such internalization leads to pronounced effects due to the drugs. As expected, we observed Nano-CUR to be more effective in reducing cell viability and clonogenicity of cervical cancer cells (Fig. 2). Furthermore, the superior functional effects of Nano-CUR was confirmed through flow cytometry by performing cell cycle and apoptosis assays. Nano-CUR was observed to be more effective in arresting cells in G1-S transition as compared to free CUR at higher concentrations (Fig. 3A). Additionally, Nano-CUR was found to be more effective in comparison to free CUR in inducing apoptosis in cervical cancer cells, wherein it induced 63–88% late apoptosis at higher concentrations (Fig. 3B).

To further verify the improved therapeutic efficacy of the Nano-CUR formulation, we generated an orthotopic mouse model of cervical cancer (Caski cells) using NSG mice. Nano-CUR treatment was more effective to inhibit the tumor growth over all other groups (Fig. 4B,C). This demonstrates that curcumin showed better therapeutic effects in its nanoformulation form (Fig. 4). Previous studies from our lab^5^ have shown that curcumin decreases the expression of the oncogenic HPV protein E7 in *in vitro* models. This led us to confirm the status of HPV oncogenic proteins E6 and E7 in NSG mice tumor tissues that were collected after the treatment. CUR and Nano-CUR treatment effectively suppressed the expression of E6 and E7 oncoproteins. Nano-CUR, however, was more efficient than free CUR in decreasing the expression levels of Ki67, a well-known marker for cell proliferation (Fig. 4D).

Furthermore, the anti-oncogenic effects of CUR and Nano-CUR were measured against smoke carcinogen, BaP. The cancer causing effects of BaP, such as increasing the expression of oncogenic entities miRNA-21 and IL-6 and decreasing the expression of the tumor suppressor miRNA-214, were mitigated by CUR and Nano-CUR. These effects were even more significant with Nano-CUR treatment (Fig. 5A–C). CUR/Nano-CUR were also effective in restoring the anti-survival pathways affected by BaP treatment through decreasing the expression of oncogenic transcription factors such as p-STAT3/5, NF-κB and β-catenin and enhancing the expression of the tumor suppressor phosphatase PTEN, besides suppressing its inactive form p-PTEN (Fig. 5D). Polycyclic aromatic hydrocarbons like BaP or its derivatives are known to increase the expression of pro-inflammatory cytokines such as IL-8 or IL-6^52^,^53^ in various organs and cancers. IL-6 mediated activation of transcription factor STAT3 (to its activated form p-STAT3) is a common oncogenic process and is one of the mechanisms through which chronic inflammation contributes to cancer development and progression^31^. Previous studies have demonstrated that an upstream enhancer that contains two STAT3 binding sites controls the gene encoding miRNA-21 and miRNA-21 expression is dependent on binding of the activated form of STAT3 (p-STAT3) to its promoter regions^31^. Moreover, these studies also show that miRNA-21 gene transcription is controlled by IL-6 and entails STAT3. The overexpression of miRNA-21 has been observed in several cancers and all of those cancers have constitutively activated STAT3 (p-STAT3)^53^,^54^. Furthermore, NF-κB has also been shown to increase miRNA-21 expression in a number of studies^55^,^56^. All of this corroborates our finding in cervical cancer that BaP treatment leads to an increase in miRNA-21 expression. Additionally, CUR and Nano-CUR restore the expression of the tumor suppressor phosphatase PTEN, a direct target of miRNA-21 (Fig. 5A,D). We also validated the expression levels of miRNA-21 and PTEN in mice tumors generated through the establishment of the orthotopic model of cervical cancer. *In situ* hybridization of the tissue slides showed that CUR and Nano-CUR were effective in abrogating the expression of miRNA-21 and thus increased expression of PTEN (Fig. 5E). CUR and Nano-CUR were also able to suppress IL-6 (Fig. 5C) and BaP-induced migration of cervical cancer cells (Fig. 6A). IL-6 is known to enhance the cellular migratory properties of cancer cells^57^,^58^. Polycyclic aromatic hydrocarbons (PAHs) such as BaP are also known to enhance cellular migration in several types of cells^39^,^56^,^58^. It would be interesting to see if BaP alone or in combination with pro-inflammatory cytokines such as IL-6 can lead to enhanced inflammation which is a precursor of oncogenesis^59^. Additionally, Nano-CUR was found to be more effective than free curcumin in suppressing nuclear translocation of β-catenin and restoring membrane bound β-catenin in Caski cervical cancer cells (Fig. 6B). Nuclear localization of β-catenin is the key initiating and driving event for transformation of normal (non-tumor) cell to tumor cell (as discussed in Results). Moreover, β-catenin is a direct target of miRNA-214, a tumor suppressor miRNA in cervical cancer (as mentioned earlier in Results).

**Figure 7:**
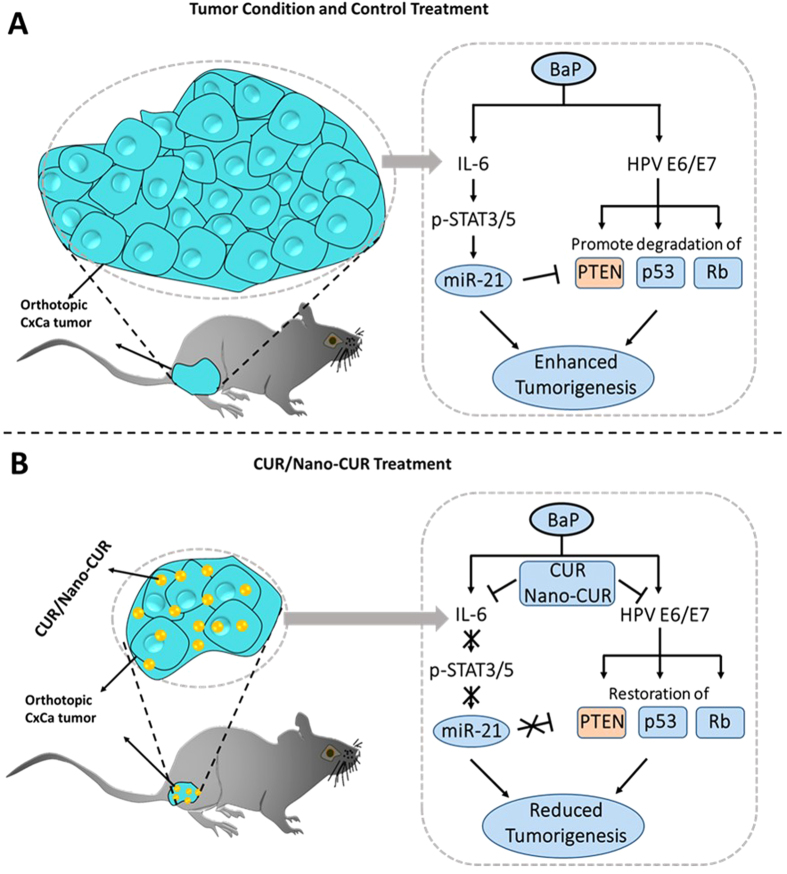
Schematic representation of CUR/Nano-CUR’s effect on the suppression of E6 and E7 HPV proteins and IL-6 pathways to reduce the tumor burden in an orthotopic cervical cancer mouse model.

In conclusion, this study clearly is an advancement of an earlier report of curcumin nanoformulation tested against cancer cells^60^. Our findings show that PLGA based Nano-CUR significantly inhibits growth of cervical cancer cells and regulates the expression of miRNAs and various oncogenic and tumor suppressor proteins associated with cervical cancer (Fig. 7). *In vivo* experiments show that Nano-CUR is efficacious in reducing the tumor burden. Therefore, Nano-CUR may be a novel chemo-preventive and therapeutic modality for the management of cervical cancer.

## Materials and Methods

### Cell Culture

Caski and SiHa cervical cancer cell lines were purchased from American Type Culture Collection (Manassas, VA). These cell lines were expanded and low passage frozen aliquots were stored in liquid nitrogen. For conducting experiments, cells were thawed and grown for <6 months. SiHa cells were maintained in Dulbecco’s Modified Eagle’s medium (DMEM) containing 4.5 g/L of glucose, 10 nM of nonessential amino acids, 100 nM of sodium pyruvate, 1x antibiotic/antimycotic (Sigma, St. Louis MO), and 10% heat-inactivated FBS (Atlanta Biologicals, Lawrenceville, GA). Caski cells were grown in Roswell Park Memorial Institute (RPMI) medium containing 10% heat-inactivated FBS and 1x antibiotic/antomycotic. All cells were incubated in a 5% CO_2_ incubator at 37 °C.

### Preparation of Nano-CUR formulation

The PLGA based CUR nanoformulation was synthesized as described previously^22^. Briefly, 90 mg of PLGA solution in 10 mL of acetone was mixed with 10 mg of CUR for 5 min. This solution was added dropwise to 20 mL of aqueous solution containing 1% wt/vol PVA and 10 mg of poly(l-lysine), over a period of 10 min on a magnetic stirrer at 800 rpm. The resulting suspension of Nano-CUR particles was stirred at room temperature for ~24 hrs to evaporate the acetone solvent completely. Larger aggregates and un-bound polymers were removed by centrifugation at 5000 rpm in an Eppendorf Centrifuge 5810 R (Eppendorf AG, Hamburg, Germany) for 10 min. Nano-CUR particles were recovered by ultracentrifugation at 30,000 rpm using a Rotor 30.50 in an Avanti J-30I Centrifuge (Beckman Coulter, Fullerton, CA, USA). Particles were washed twice and then freeze-dried using the Labconco Freeze Dry System (−48 °C, 133 × 10^–3^ mBar; Labconco, Kansas City, MO). Control PLGA nanoparticles were similarly prepared by dissolving polymer in organic solvent without CUR, with the rest of the method being the same.

### Cellular uptake

To determine the uptake and internalization efficacy of Nano-CUR, we utilized fluorescent microscopy and flow cytometry. For qualitative assessment, Caski and SiHa cells (2 × 10^5^ cells/well in 2 mL media) were plated in 6-well plates and allowed to attach overnight. Cells were then treated with 5, 10, and 20 μM CUR or Nano-CUR and their vehicle controls (DMSO and PLGA NPs, respectively) for 6 hrs. After 6 hrs, cells were washed with PBS twice and replaced with phenol-red free medium. Live adherent cells were examined under a fluorescent microscope (Nikon Eclipse Ti Microscope, Melville, NY) and images were captured at 200X. For quantitative measurement, cells (2 × 10^5^ cells per well in 2 mL media) were plated in 6-well plates and allowed to attach overnight. Cells were then treated with 5, 10, and 20 μM CUR or Nano-CUR and their vehicle controls (DMSO and PLGA-NPs, respectively) for 6 hrs. After 6 hrs, cells were washed with PBS, trypsinized, and collected in 2 mL media. Further, these cells were centrifuged, washed with PBS twice, and collected in 2 mL phenol-red free medium for flow cytometry analysis. About 10,000 cells in medium were injected into an Accuri C6 Flow Cytometer (BD Biosciences, San Jose, CA, USA) to determine the fluorescence levels in the FL1 channel (488 excitation, Blue laser, 530 ± 15 nm, FITC/GFP). FL1 channel measures the green fluorescence generated by the curcumin or Nano-CUR.

### Cell proliferation assay

The effect of CUR and Nano-CUR on cell growth was determined by cell proliferation assay using CellTiter96 Aqueous One Solution (MTS) reagent (Promega, Madison, WI). Briefly, cervical cancer cells (5000 cells/well) were plated in 100 μL DMEM/RPMI medium in 96-well tissue culture plates and incubated overnight. CUR was dissolved in DMSO and diluted in cell culture media. The cells were treated with varying concentrations of CUR/Nano-CUR or equivalent amounts of vehicle controls (DMSO/PLGA) for 48 hrs. Cell proliferation was determined by adding 20 μL of MTS reagent, incubating for 2 hrs at 37 °C and measuring the absorption at 490 nm using a SPECTRA Max Plus Plate Reader (Molecular Devices, Sunnyvale, CA). The percent proliferation in CUR/Nano-CUR treated cells was determined by normalizing the cells with no treatment (considered as 100%).

### Colony forming assay

For this assay, cervical cancer cells were seeded at 500 cells per well in 2 mL media in 6-well plate and allowed for 24 hrs to attach. After that, cells were treated with concentrations of 2.5, 5, and 10 μM CUR/Nano-CUR for an additional 12–14 days. The cells were washed, fixed in cold methanol, and stained with hematoxylin. Visible colonies (~50 cells) were counted manually and reported as the ratio of the number of colonies in treated cells divided by the number of colonies in the vehicle (DMSO/PLGA) control.

### Cell cycle analysis

Cell cycle arrest was analyzed by the Telford method. Caski and SiHa cervical cancer cells (1 × 10^6^) were plated in a 100 mm dish and allowed to attach overnight. The following day, cells were exposed to either 10 or 25 μM CUR, Nano-CUR or equivalent amounts of controls for 24 hrs, trypsinized, washed, fixed with 70% ethanol, stored at 4 °C for an hr, stained with propidium iodide (Sigma-Aldrich, St. Louis, MO; 50 μg in 1 mL Telford reagent) in the dark for 4 hrs at 4 °C and analyzed by an Accuri C6 Flow Cytometer in FL2 channel.

### Annexin V-7AAD staining

Detection of apoptosis was determined by using Annexin V-7AAD staining. Caski and SiHa cervical cancer cells (1 × 10^6^) were plated in a 100 mm dish and allowed to adhere overnight. The next day, cells were treated with 10, 20 and 25 μM CUR, Nano-CUR or equivalent amounts of controls for 24 hrs. At the end of indicated time, both adherent and floating cells were collected and stained with Annexin V and 7-AAD (BD Biosciences) at 5 μL of each/100 μL of cell suspension. Cells were incubated for 20 min in the dark at room temperature and analyzed with an Accuri C6 Flow Cytometer in FL2 and FL3 channels.

### Orthotopic model of cervical cancer in NSG mice

Six-week-old female NSG mice (Cancer Research Animal Core, UTHSC, Memphis, TN) were used to generate an orthotopic model of cervical cancer. The mice were maintained in a pathogen-free environment and all procedures were carried out as approved by the UTHSC Institutional Animal Care and Use Committee (UTHSC-IACUC). All the procedures and methods were carried out in “accordance” with the approved guidelines of UTHSC-IACUC. Briefly, Caski cells (4 × 10^**6**^ cells/per mouse) were dispersed in 100 μL 1X PBS and 100 μL Matrigel (BD Biosciences) and injected directly into the cervix of the mice. The animals were periodically monitored for tumor development and the tumor volume was measured from day 5 after injection using a digital Vernier caliper. When the tumor volume reached ~200 mm^3^, CUR, Nano-CUR (100 μg/mice) and their respective vehicle controls (PBST and PLGA NPs, respectively) were injected intra-tumorally. The tumor volume was calculated using the ellipsoid volume formula: tumor volume (mm^3^) = *π***/**6 × *L* × *W* × *H*, wherein *L* is length, *W* is width, and *H* is height. The tumor was regularly monitored and allowed to grow until the tumor burden reached a maximum volume of 1100 mm^3^. At the time of sacrifice, the mice tumors were removed, fixed in formalin, embedded in paraffin, and sliced into 5 micron sections for further processing and analysis.

### Reverse transcription quantitative real-time polymerase chain reaction (qRT-PCR)

Caski cervical cancer cells were seeded in a 6-well plate, allowed to attach, and treated with varied concentrations of CUR/Nano-CUR and their vehicle controls (DMSO/PLGA) and BaP in their respective combinations for 4 days. Total RNA was extracted from treated cells using TRIzol reagent (Invitrogen, Life Technologies, Grand Island, NY). The integrity of the RNA was measured with an RNA 6000 Nano Assay kit and 2100 Bioanalyzer (Agilent Technologies, Santa Clara, CA). For miRNA detection, 100 ng total RNA was reverse transcribed into cDNA using specific primers designed for miRNA analysis (Applied Biosystems, Foster City, CA). The miRNA expression levels were determined by qRT-PCR using the Taqman PCR master mixture (no AmpErase UNG) and specific primers designed for detection of mature miRNAs (Applied Biosystems). The expression of miRNA was normalized with the expression of endogenous control, U6snRNA.

### IL-6 measurement

Caski cervical cancer cells were treated using modified culture medium (RPMI-1640 + 2% FBS). Briefly, after treatment with BaP alone or in combination (BaP and CUR/Nano-CUR) for 48 hrs, culture medium was centrifuged at 10,000 g for 10 min at 4 °C. OD was measured at 450 nm. IL-6 (eBioscience, San Diego, CA, USA) measurement was performed as per manufacturer’s instructions. Quantification of IL-6 was performed by standard curve prepared by recombinant human IL-6 (provided with the kit) and normalized with live cells. All experiments were performed in triplicate and repeated at least three times.

### Migration assay

Cell migration assay was performed in Corning’s 96-well HTS transwell as per manufacturer’s instructions with minor modifications. Briefly, Caski cells (50,000 cells/well) were seeded in upper chambers of the plate containing serum-free culture medium. Cells were further treated with BaP alone, or in combination (BaP and CUR/Nano-CUR) for 18 hrs. Cells were allowed to migrate from upper chamber (5% FBS) towards lower chamber which has 10% FBS. Cells in the upper chamber were completely removed by cotton swab. Cells were fixed with 4% paraformaldehyde prepared in PBS for 30 min. These cells were stained with Giemsa stain and migrated cells were observed by using a light microscope. Migrated cells were counted in six random fields of view and the experiments were performed in triplicate.

### Western blotting

Actively growing Caski cervical cancer cells were used for Western blot analysis. Briefly, Caski cells were washed with ice-cold phosphate buffer saline (PBS) and lysed in 2X radioimmunoprecipitation assay (RIPA) buffer after the respective treatments. Protein content was analyzed using Nanodrop 2000 (Thermo Scientific, Wilmington, DE), and equivalent amount of protein samples were electrophoretically separated on 4–20% sodium dodecyl sulfate polyacrylamide gel electrophoresis (SDS-PAGE) gels, and transferred to a polyvinylidene difluoride (PVDF) membrane (Bio-Rad Laboratories, Hercules, CA). Following blocking with 5% bovine serum albumin (BSA; 5 mL for one hour), the membranes were probed overnight at 4 °C for various proteins using specific primary antibodies (all from Cell Signaling Technologies, Danvers, MA). The western blots were incubated with HRP-labeled secondary antibody and the protein bands were developed using Lumi-Light Plus chemiluminescent reagent (Roche, Indianapolis, IN).

### Immunofluorescence and Confocal Microscopy

Immunofluorescence staining was performed to determine the effect of BaP on the nuclear translocation of β-catenin. 80,000 Caski cells were seeded overnight in 4-well chamber slides (Nalgene Nunc Intl.., Rochester, NY), then treated with BaP alone and in combination with CUR/Nano-CUR for 24 hrs. Subsequently, they were fixed and processed for immunostaining. Briefly, cells were fixed in 2% para-formaldehyde, permeabilized, blocked and then incubated with primary antibody, anti β-catenin Ab (Cell Signaling Technologies). Following washing, cells were incubated with Cy3 labeled secondary antibody (Jackson Immunoresearch Labs, Westgrove, PA). After further washing, the cover slips were mounted on glass slides using aqueous anti-fade medium (Vector Laboratories, Burlingame, CA). Slides were examined under a laser confocal microscope (Nikon Corporation).

### *In situ* hybridization for miRNA-21

*In situ* hybridization was conducted according to the manufacturer’s protocol for FFPE tissues of control and treated orthotopic mice to detect the expression of miRNA-21 using a Biochain kit (Biochain, San Francisco, CA). DIG-labeled LNA oligonucleotides (Exiqon, Woburn, MA) were used for overnight hybridization at 50 °C. The staining was carried out as per Exiqon user manual instructions. Briefly, after deparaffinization, specimens were fixed in 4% para-formaldehyde in DEPC-PBS for 20 min and subjected to digestion using 2X standard saline citrate and 0.1% Triton-X for the next 25 min. Slides were pre-hybridized with pre-hybridization solution for 4 hrs at 48 °C followed by hybridization of the slides with hybridization buffer and probe (Digoxigenin labeled) at 45 °C overnight. After stringent washing of slides with various grades of standard saline citrate, the slides were blocked using 1X blocking solution provided in the kit. The tissues were subsequently incubated overnight with the AP-conjugated anti-digoxigenin antibody. The slides were washed twice for 5 min with 1X Alkaline Phosphatase buffer. The final visualization was carried out with NBT/BCIP overnight followed by nuclear fast red counterstaining. The slides were mounted, imaged and analyzed under ScanScope® XT/ XT2 system (Aperio, Vista, CA). All the reagents used for the assay were provided in the kit from Biochain.

### Immunohistochemical analysis of cervical orthotopic tumors

IHC analysis for HPV E6/E7 proteins, Ki67 and PTEN on formalin fixed, paraffin embedded orthotopic mouse tumors (5 micron sections) was performed. Briefly, the tumor tissues were deparaffinized, rehydrated, treated with 0.3% hydrogen peroxide and processed for antigen retrieval using a heat-induced technique. Following blocking with background sniper (Biocare Medical, Concord, CA), the samples were processed for staining with E6 (Abcam, Cambridge MA), E7 (Invitrogen), Ki67 and PTEN antibodies (Cell Signaling Technologies). The expression of these proteins was detected using a MACH 4 Universal HRP Polymer detection kit (Biocare Medical) and 3,39-diaminobenzidine (DAB substrate kit, Vector Laboratories, Burlingame, CA). The slides were counterstained with hematoxylin, dehydrated, mounted with VectaMount (Vector Laboratories) and visualized using an Olympus BX 41 Microscope (Olympus Corporation, Japan).

### Statistical analysis

Values were processed using Microsoft Excel 2007 software and presented as mean ± standard error of the mean (S.E.M.). Statistical analyses were performed using an unpaired, two-tailed student t-test. The level of significance was set at *p < 0.05. All graphs were plotted using Origin 6.1 software.

## Additional Information

**How to cite this article**: Zaman, M. S. *et al*. Curcumin Nanoformulation for Cervical Cancer Treatment. *Sci. Rep*. **6**, 20051; doi: 10.1038/srep20051 (2016).
